# Supplementary material for: Attractiveness of thermally different, uniformly black targets to horseflies: Tabanus tergestinus prefers sunlit warm shiny dark targets
Source: R Soc Open Sci. 2019 Oct 23;6(10):191119. doi: 10.1098/rsos.191119 (PMC6837212; doi:10.1098/rsos.191119)
Supplement: Electronic Supplementary Materials [file rsos191119supp1.doc]

**Electronic Supplementary Materials**

for

**Attractiveness of thermally different, uniformly black targets to horseflies: *Tabanus tergestinus* prefers sunlit warm shiny dark targets**

Gábor Horváth*, Ádám Pereszlényi, Tímea Tóth, Szabolcs Polgár and Imre M. Jánosi

*correspondig author: Environmental Optics Laboratory, Department of Biological Physics, ELTE Eötvös Loránd, University, H-1117 Budapest, Pázmány sétány 1, Hungary, e-mail: gh@arago.elte.hu

This file contains the following:

Supplementary Materials and Methods

Calibration of the Thermocamera

Supplementary Results

Reflection-polarization characteristics of the warm and cold barrels

Thermal characteristics of the warm and cold barrels

Schlieren imagery of the warm/cold air next to the warm/cold barrels

Attractiveness of the warm and cold barrels to tabanids

Supplementary Tables S1, S2, S3

Supplementary Figures S1, S2, S3, S4, S5, S6, S7

**Supplementary Materials and Methods**

**Calibration of the Thermocamera**

In order to demonstrate that our thermocamera (VarioCAM®, Jenoptik Laser Optik Systeme GmbH, Jena, Germany, with a nominal precision of ±1.5 ^o^C) really measures the surface temperature correctly and precisely, we have measured the surface temperature of a cold and warm black coffe between 3 and 55 ^o^C (Supplementary Figure S1) and a hot reddish tile between 27 and 67 ^o^C (Supplementary Figure S2) by our thermocamera and contact thermometer (GAO Digital Multitester EM392B 06554H, EverFlourish Europe Gmbh., Friedrichsthal, Germany, with a nominal precision of ±1 ^o^C). Supplementary Figure S3 displays the graph of surface temperatures measured by our thermocamera and contact thermometer.

These data provide experimental evidence that our thermocamera can measure the surface temperature with an accuracy of about ±1.5 ^o^C.

The small differences between the temperatures measured simultaneously by the contact thermometer and the thermocamera are due to many factors: (i) The metal tip of the contact thermometer conducted some heat flux from/to the air to/from the tiny target volume to be measured. (ii) This tip was immersed in the coffee slightly (circa 1 mm) below the coffee-air interface, thus it could not measure the temperature of the coffee surface, which was measured by the thermocamera. (iii) At higher temperatures, the enhanced evaporation strongly cools the fluid surface (skin layer) resulting in systematically increasing differences between the two temperature values. (iv) The majority of the tip of the contact thermometer was in the air layer directly above the tile’s surface. Obviously, the contact thermometer could not be pressed into the uppermost thin (20-50 μm) surface layer of the tile from which the electromagnetic radiation was emitted.


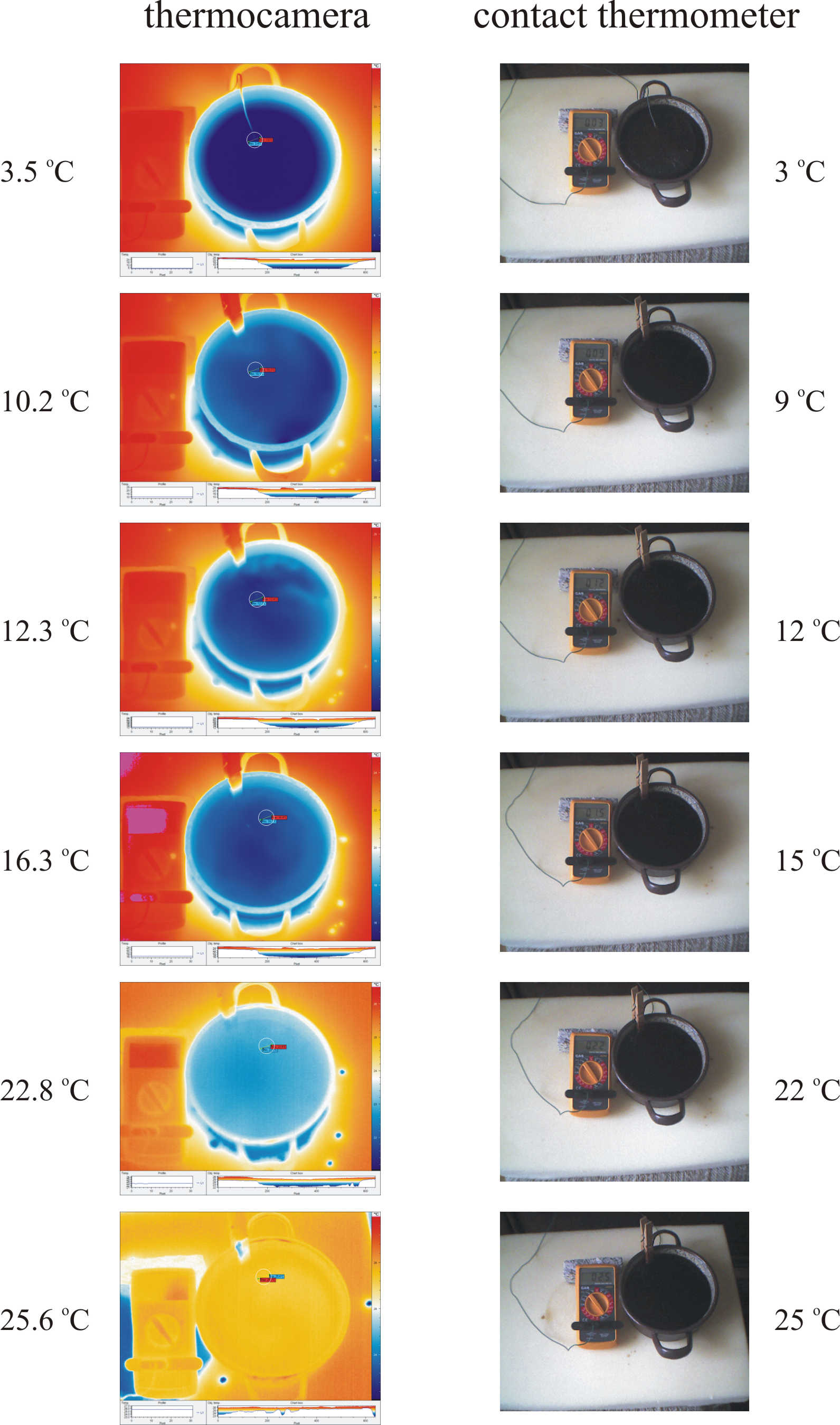


**Supplementary Figure S1**: Thermograms of a coffee surface with different temperatures ranging from 3 to 25 ^o^C measured simultaneously by our thermocamera and contact thermometer (photos and thermograms are taken by Gábor Horváth and Imre Jánosi).


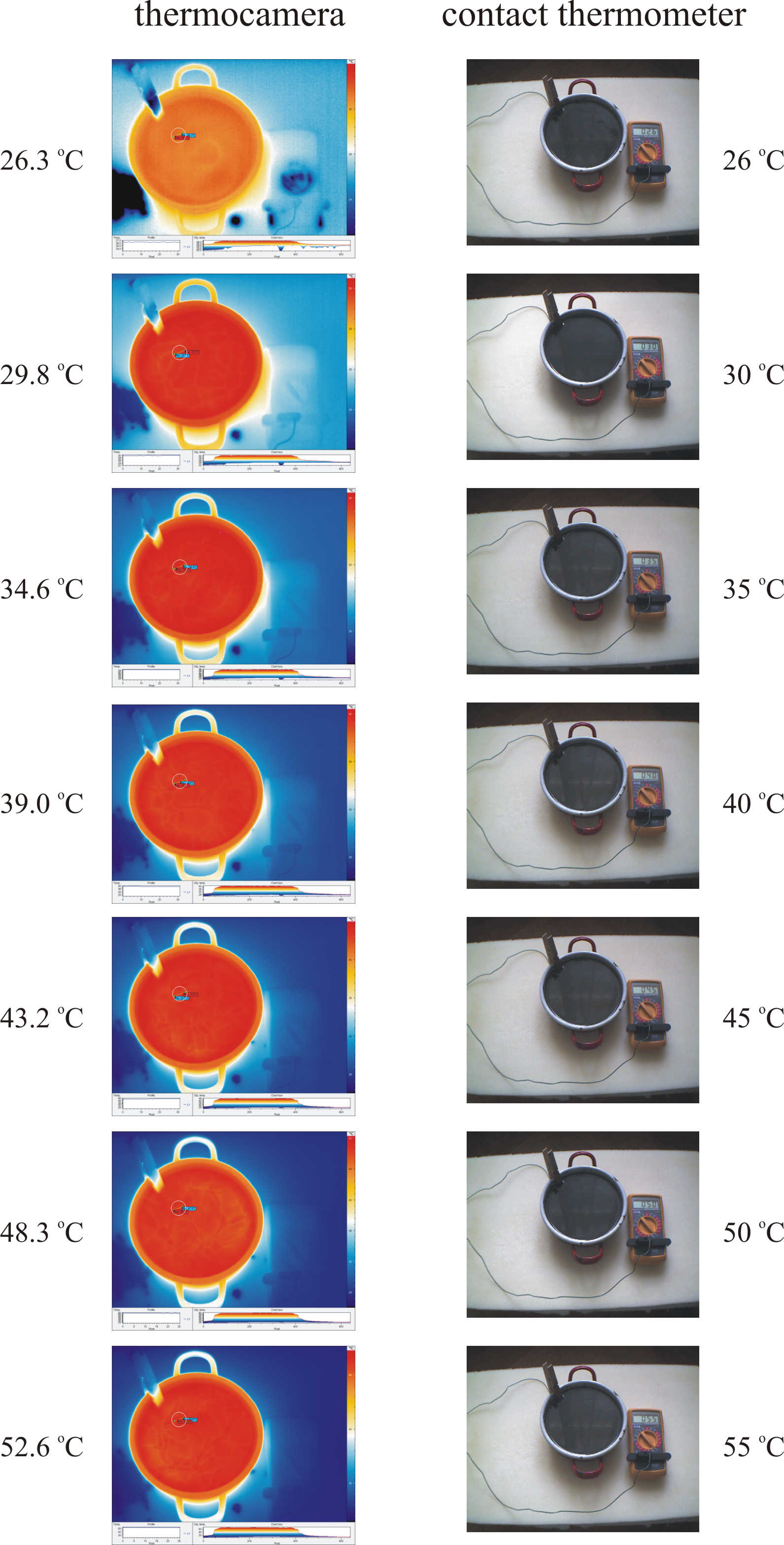


**Supplementary Figure S1 continued**: Thermograms of a coffee surface with different temperatures ranging from 26 to 55 ^o^C (photos and thermograms are taken by Gábor Horváth and Imre Jánosi).


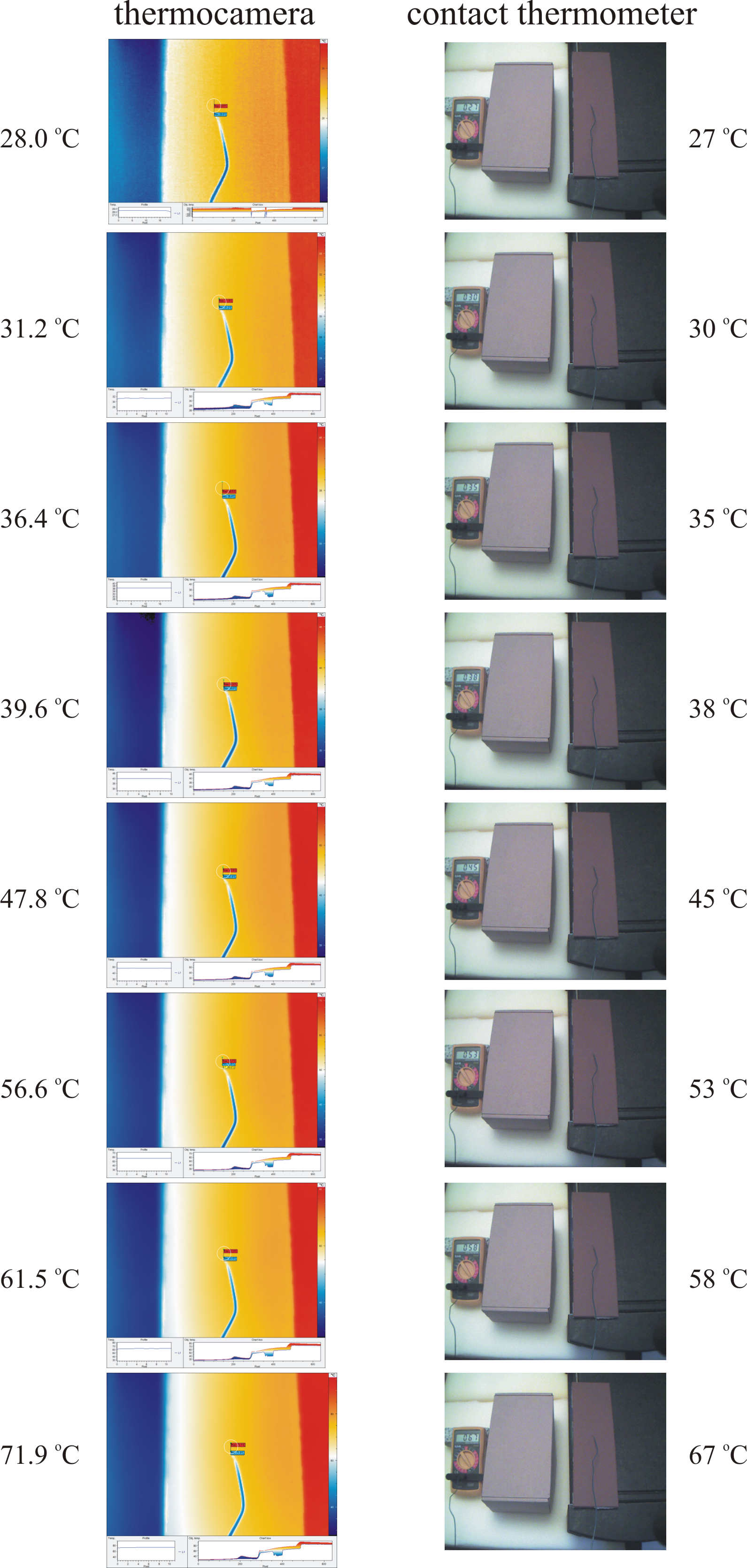


**Supplementary Figure S2**: Thermograms of the surface of a reddish tile with different surface temperatures ranging from 27 to 67 ^o^C measured simultaneously by our thermocamera and contact thermometer (photos and thermograms are taken by Gábor Horváth and Imre Jánosi).


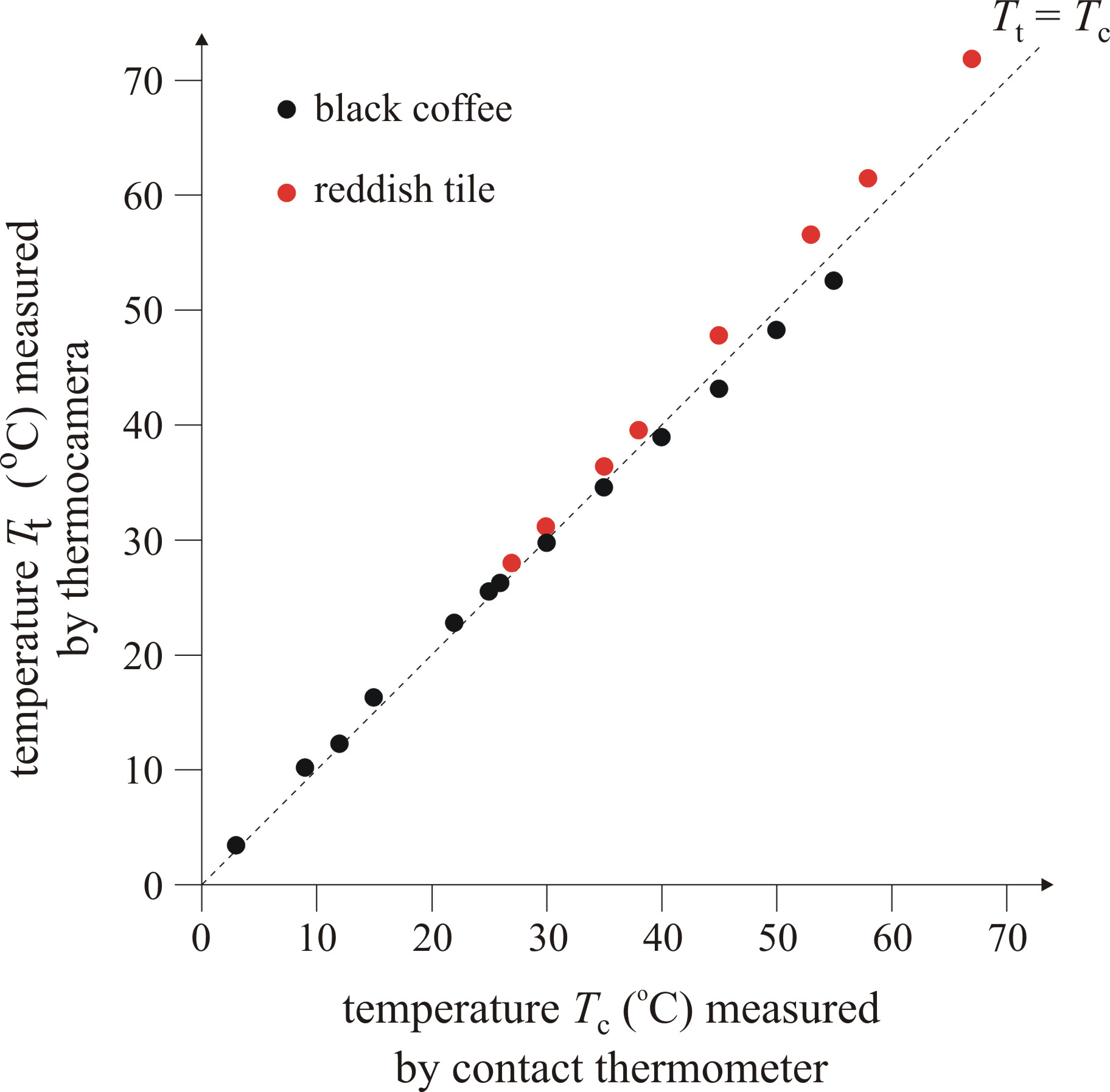


**Supplementary Figure S3**: Graph of surface temperatures measured simultaneously by our thermocamera (*T*_t_: vertical axis) and contact thermometer (*T*_c_: horizontal axis) (see Supplementary Figures S1 and S2). The dashed line corresponds to *T*_t_ = *T*_c_.

**Supplementary Results**

**Reflection-polarization characteristics of the warm and cold barrels**

Supplementary Figure S6 shows the photographs and the patterns of the degree of linear polarization *d* and the angle of polarization α of light reflected from the solar and antisolar sides of the warm and cold black barrels used in the choice experiments measured by imaging polarimetry in the green (550 nm) part of the spectrum. It is clearly seen in Supplementary Fig. S6 that the optical characteristics of the warm and cold barrels were the same. The barrel’s surface reflected maximally polarized (i.e. with the highest degree of polarization *d*_max_ ≈ 100 %) light when the angle of reflection was equal to the Brewster’s angle θ_Brewster_ = arctan(*n*=1.5) = 56.3^o^ measured from the normal vector of the surface, where *n* = 1.5 is the refractive index of the barrel’s plastic material. Tabanids landed exclusively on the curved neck and bottom as well as the vertical shell of the barrels, since the highly polarized reflected light is very attractive to blood-seeking female tabanids, independently of the direction of polarization (Horváth 2014). The directions of polarization of reflected light were approximately vertical at the periphery of the vertical shell of the barrels, while they were nearly horizontal in the middle vertical zone of the shell. The horizontal surface of the barrel’s cap reflected horizontally polarized light with maximal degree of polarization *d*_max_ ≈ 100 % at the Brewster’s angle. Tabanids never landed on this cap, since water-seeking female and male tabanids are attracted to horizontally polarized light only if it is reflected from a surface being in the ground level and elevated horizontal reflectors are unattractive to tabanids (Egri *et al*. 2013a,b, Horváth 2014). In the red (650 nm) and blue (450 nm) spectral ranges, the reflection-polarization characteristics of the warm and cold barrels were practically the same as those in the green (550 nm) range (Supplementary Figure S6).

**Thermal characteristics of the warm and cold barrels**

Figure 2 shows the thermograms of the solar and antisolar sides of the warm and cold black barrels used in experiment 1 measured in sunshine at 9:50 h (= local summer time = UTC + 2 h) on 10 July 2018. In Fig. 2 it is clearly seen that in sunshine the warmest was always the solar side of the warm barrel, the coldest was the antisolar side of the cold barrel, while the surface temperatures of the antisolar side of the warm barrel and the solar side of the cold barrel were inbetween, the former being the warmer. Hence, in sunshine the relation among the surface temperatures *T* of the solar and antisolar sides of the warm and cold barrels was: *T*_warm_(solar) > *T*_warm_(antisolar) > *T*_cold_(solar) > *T*_cold_(antisolar). This is also corroborated with the results presented in Fig. 1. When the sun was rarely occluded by clouds for shorter (< 30 minutes) periods during the experiments, depending on the duration of sun occlusion, *T* of the solar and antisolar side of the warm barrel became lower (solar side: with 10-15 ^o^C, antisolar side: with 5-10 ^o^C) than that in sunshine (Fig. 1). In these cases the temperature drop of both sides of the cold barrel was small (< 5 ^o^C). However, also in such sun-occluded situations the relation among the temperatures of the different sides of the warm and cold barrels remained the same as in sunshine: *T*_warm_(solar) > *T*_warm_(antisolar) > *T*_cold_(solar) > *T*_cold_(antisolar) (Fig. 1). Thus, during the experiments always four test surfaces (sides of the barrels) with different temperatures and uniform optical characteristics were offered for flying tabanids.

At a given point of time, the temperature *T* slighly changed spatially on the barrel’s surface, being always the lowest and highest on the bottom and top, respectively. This gradient resulted from the different angles of incidence of sunlight in the case of both the warm (air-filled) and cold (water-filled) barrels. On the other hand, in the water-filled barrel, the density of warmer water was smaller than that of colder one (if *T* > 4 ^o^C). Due to this small spatial variation, *T* was averaged (Fig. 1) in the rectangles on the barrel’s shells displayed in Fig. 2C,D.

In experiment 1, the difference in the surface temperature *T* between the same (solar or antisolar) side of the warm and cold barrels slowly decreased as a function of time because of the gradual warming up of the iceakkus in the water of the cold barrel (Fig. 1A). In experiments 2-4, the same warming up of the cold barrel occured until the iceakkus were refreshed in it at about half-time. After this refreshing, *T* of the cold barrel suddenly dropped, then it began again slowly warming up (Fig. 1B).

**Schlieren imagery of the warm/cold air next to the warm/cold barrels**

Supplementary Figure S5 shows the photographs and thermograms of the hot (*T* = 70.2 ± 1.1 ^o^C), warm (*T* = 45.5 ± 0.5 ^o^C) and cold (*T* = 8.5 ± 1.7 ^o^C) plastic flasks used for Schlieren imagery and modelling well the thermal characteristics of the warm/cold barrels used in the choice experiments. We used Schlieren imagery, because a thermocamera cannot measure and visualize the temperature of the air around the warm/cold flask. Figure 3 displays the Schlieren images of the air flow next to the hot (*T* = 70 ^o^C), warm (*T* = 45 ^o^C) and cold (*T* = 8 ^o^C) flask when the air was calm (windless) or turbulent (windy, because the flask was very weakly blown by mouth from 1 m). It is clearly seen that the warm flask warmed up the boundary layer (i.e. the air next to the flask’s wall) and this warm air streamed upward due to its smaller density than that of the surrounding air of 20 ^o^C room temperature. On the other hand, the cold flask cooled down the boundary layer and this cold air streamed downward due to its larger density than that of the surrounding air (Fig. 3). In calmness (under windless conditions), the thickness of the upward flowing warm and downard streaming cold boundary layer of the warm and cold flask was 1-2 cm. However, under turbulent (windy) conditions, the warm and cold air mixed turbulently, thus an elongated warm/cold turbulent train of several centimeters was formed beyond the warm/cold flask toward the wind direction (Fig. 3).

**Attractiveness of the warm and cold barrels to tabanids**

Supplementary Figure S7A shows the total abundance number *M* of time *t* spent by tabanids on the warm and cold barrels in experiments 1-5. *N* decreased monotonously and steeply with increasing *t*. The most frequent *t*-values were shorter than 5 s. 440 tabanids stayed on one of the barrels for periods not longer than 5 s. On the other hand, the number *N* of tabanid landings on either of the barrels for staying periods 5 s < *t* ≤ 10 s and 10 s < *t* ≤ 15 s was only 200 and 100, respectively. *N* was smaller than 35 for periods τ < *t* ≤ τ + 5 s, if τ was larger than 40 s.

Supplementary Figure S7B-C represents the abundance number *M* of *t* spent by tabanids on the solar side of the warm and cold barrel in experiments 1-2. On the warm barrel, *N* decreased tendentiously with increasing *t*, the most frequent *t*-values were 5 s < *t* ≤ 10 s, *N* suddenly dropped at *t* = 40 s and remained smaller than 13 for *t* > 40 s (Supplementary Fig. S7B). On the cold barrel, *N* decreased steeply and quasi monotonously with increasing *t*, the most frequent *t*-values were shorter than 5 s, and *N* was smaller than 20 for *t* > 10 s (Supplementary Fig. S7C).

Supplementary Figure S7D-G displays the abundance number *M* of *t* spent by tabanids on the solar and antisolar sides of the warm and cold barrels in experiments 3-5. On the solar side of the warm barrel, *N* decreased slowly and tendentiously with increasing *t*, the most frequent *t*-values were 5 s < *t* ≤ 10 s, and *N* had a striking local maximum (*N* = 19) for 35 s < *t* ≤ 40 s (Supplementary Fig. S7D). On the antisolar side of the warm barrel, *N* decreased steeply and quasi monotonously with increasing *t*, the most frequent *t*-values were shorter than 5 s, and *N* was smaller than 10 for *t* > 35 s (Supplementary Fig. S7F). On both (solar and antisolar) sides of the cold barrel, *N* decreased steeply and tendentiously with increasing *t*, the most frequent *t*-values were shorter than 5 s, and *N* was smaller than 10 for *t* > 20 s (Supplementary Fig. S7E,G). On the antisolar side of the cold barrel, *t*-values larger than 10 s practically did not occur (Supplementary Fig. S7G).

**Supplementary Table S1:** Surface temperatures *T* (^o^C) of a white, a brown and a black horse measured by thermography on an area and along a nearly horizontal and vertical line under sunlit (illuminated by direct sunlight) and shady (sun was behind a cloud) conditions. <*T*>: average temperature, Δ*T*: standard deviation of *T*.

| **colour** | **area** | **along a horizontal line** | | | | **along a tilted line** | | | |
| --- | --- | --- | --- | --- | --- | --- | --- | --- | --- |
|  | **<*T*>±Δ*T*** | ***T*_max_** | ***T*_min_** | ***T*_max_-*T*_min_** | **<*T*>±Δ*T*** | ***T*_max_** | ***T*_min_** | ***T*_max_-*T*_min_** | **<*T*>±Δ*T*** |
| **sunlit** | | | | | | | | | |
| **white,**  **black** | 36.4±1.2,  43.1±3.2 | 43.3 | 38.2 | 5.2 | 40.7±1.8 | 42.3 | 34.3 | 8.0 | 36.0±1.9 |
| **brown** | 42.0±0.8 | 44.0 | 41.6 | 2.5 | 42.7±0.9 | 44.6 | 38.5 | 6.1 | 41.1±1.5 |
| **black** | 44.4±3.4 | 51.5 | 47.7 | 3.8 | 49.1±1.3 | 50.9 | 37.5 | 13.4 | 40.2±4.7 |
| **shady** | | | | | | | | | |
| **white** | 37.3±0.4 | 38.2 | 36.6 | 1.6 | 37.3±0.3 | 37.7 | 36.7 | 1.0 | 37.2±0.7 |
| **brown** | 35.4±0.5 | 35.8 | 34.0 | 1.7 | 34.7±0.5 | 36.0 | 35.4 | 0.5 | 35.6±0.5 |
| **black** | 37.2±0.5 | 37.1 | 36.4 | 0.6 | 36.8±0.2 | 37.4 | 36.6 | 0.9 | 36.9±0.3 |

**Supplementary Table S2:** Total numbers *N* of landings and average time <*t*> ± standard deviation (second) per individual spent on the solar and antisolar sides of the warm and cold barrels in experiments 1-2 and 3-5 (Figs. 5 and 6).

| **barrel** | **warm barrel** | | | | **cold barrel** | | | |
| --- | --- | --- | --- | --- | --- | --- | --- | --- |
| **side of barrel** | **solar** | **antisolar** | **solar** | **antisolar** | **solar** | **antisolar** | **solar** | **antisolar** |
|  | ***N*** | ***N*** | ***<t*> (s)** | ***<t*> (s)** | ***N*** | ***N*** | ***<t*> (s)** | ***<t*> (s)** |
| **experiments**  **1 + 2** | 400 | - | 61±78 | - | 337 | - | 22±46 | - |
| **experiments 3 + 4 + 5**  **sum** | 299 | 254 | 65±83 | 51±115 | 179 | 109 | 22±48 | 4±9 |
|  | 553 | | 58±99 | | 288 | | 15±39 | |

**Supplementary Table S3**: Results of the statistical analysis for *N*_warm_(solar), *N*_warm_(antisolar), *N*_cold_(solar), *N*_cold_(antisolar) and *t*_warm_(solar), *t*_warm_(antisolar), *t*_cold_(solar), *t*_cold_(antisolar) comparisons using the data of Supplementary Table S2. *N* = number of landings on the different barrel’s surface, *t* = time spent by the alighted individual tabanids on the different barrel’s surface. p > 0.05: n.s. = not significant, 0.01 < p < 0.05: *, 0.001 < p < 0.01: **, p < 0.001: ***

| **A** | ***N*_warm_(solar)** | ***N*_warm_(antisolar)** | ***N*_cold_(solar)** | ***N*_cold_(antisolar)** |
| --- | --- | --- | --- | --- |
| ***N*_warm_(solar)** | - | 0.0917 | p < 0.0001 | p < 0.0001 |
| ***N*_warm_(antisolar)** | 0.0917  n.s. | - | 0.00255 | p < 0.0001 |
| ***N*_cold_(solar)** | p < 0.0001  *** | 0.00255  ** | - | 0.000176 |
| ***N*_cold_(antisolar)** | p < 0.0001  *** | p < 0.0001  *** | 0.000176  *** | - |
|  | | | | |
| **B** | ***t*_warm_(solar)** | ***t*_warm_(antisolar)** | ***t*_cold_(solar)** | ***t*_cold_(antisolar)** |
| ***t*_warm_(solar)** | - | 0.00141 | p < 0.0001 | p < 0.0001 |
| ***t*_warm_(antisolar)** | 0.00141  ** | - | p < 0.0001 | p < 0.0001 |
| ***t*_cold_(solar)** | p < 0.0001  *** | p < 0.0001  *** | - | p < 0.0001 |
| ***t*_cold_(antisolar)** | p < 0.0001  *** | p < 0.0001  *** | p < 0.0001  *** | - |


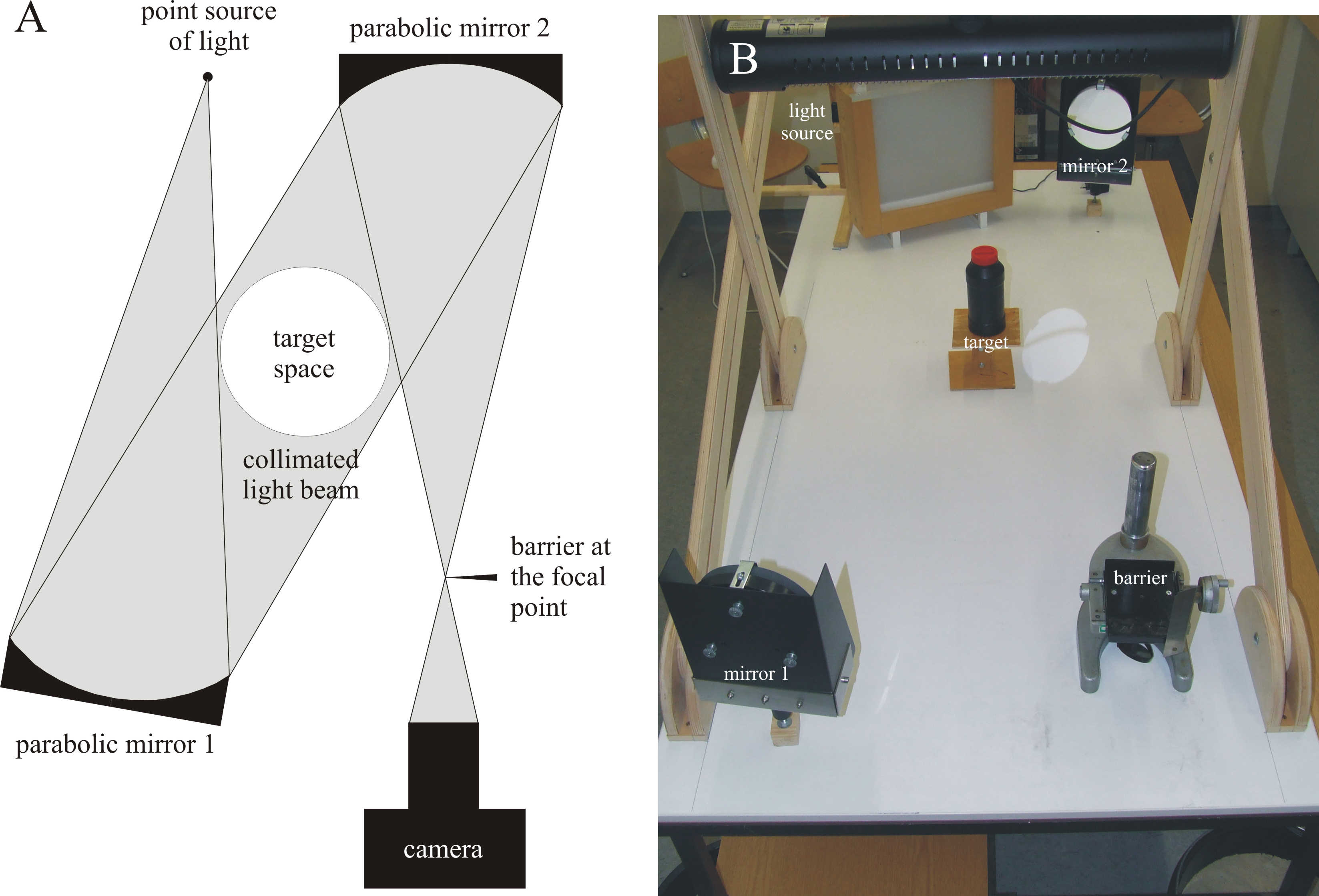


**Supplementary Figure S4:** Schematic drawing (A) and photograph (B) of the Schlieren imaging equipment used to visualize the spatiotemporal changes of the refractive index of the air next to the cold/warm flask modelling the cold/warm barrels used in our field experimnets.


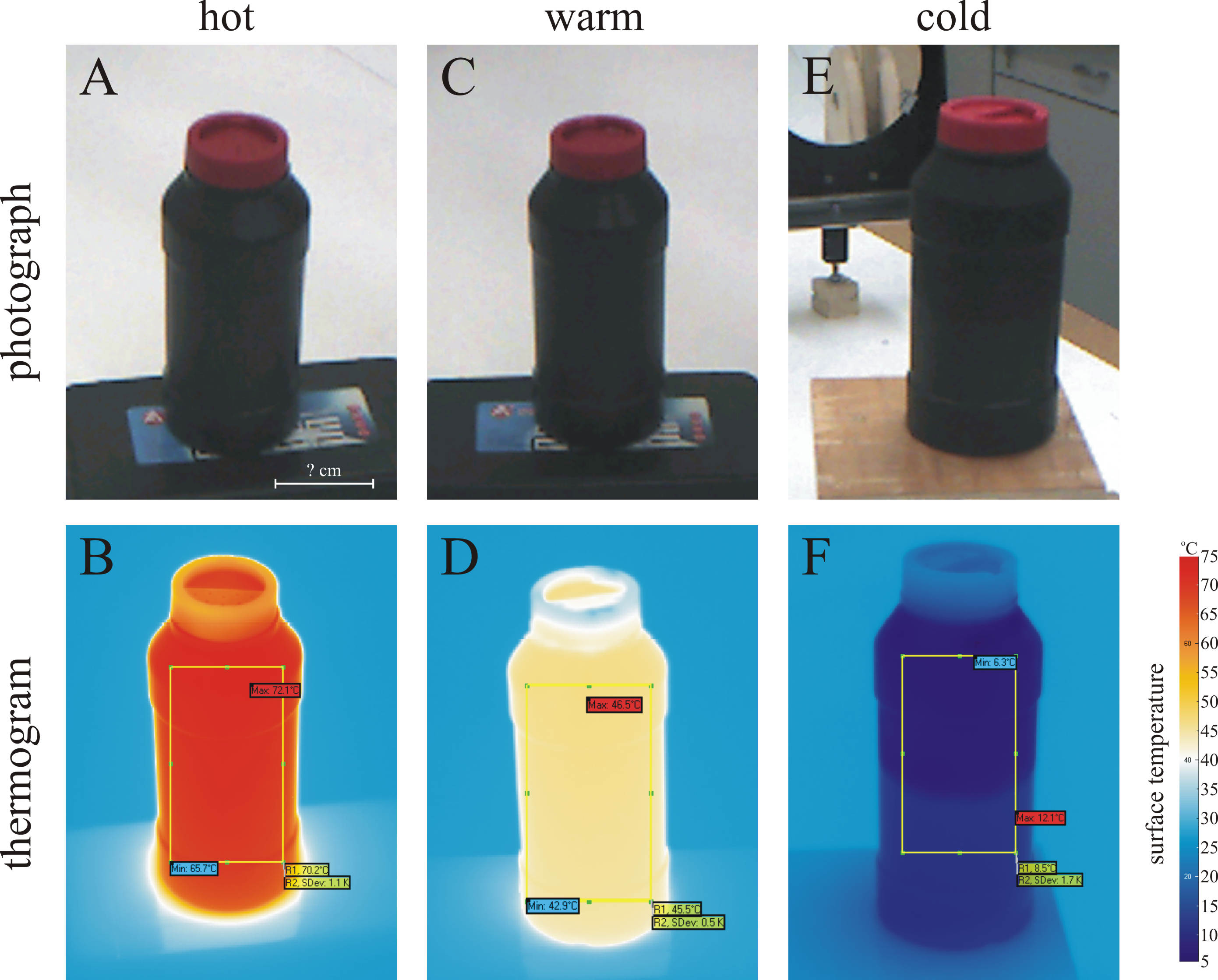


**Supplementary Figure S5:** Photographs (A, C, E) and thermograms (B, D, F) of the hot (A, B; *T* = average ± standard deviation = 70.2 ± 1.1 ^o^C), warm (C, D; *T* = 45.5 ± 0.5 ^o^C) and cold (E, F; *T* = 8.5 ± 1.7 ^o^C) plastic flasks used for the Schlieren imaging. These flasks modelled well the thermal characteristics of the warm/cold barrels used in the choice experiments. The rectangles on the flask’s shells show the areas where the surface temperature *T* was averaged (see Fig. 1).


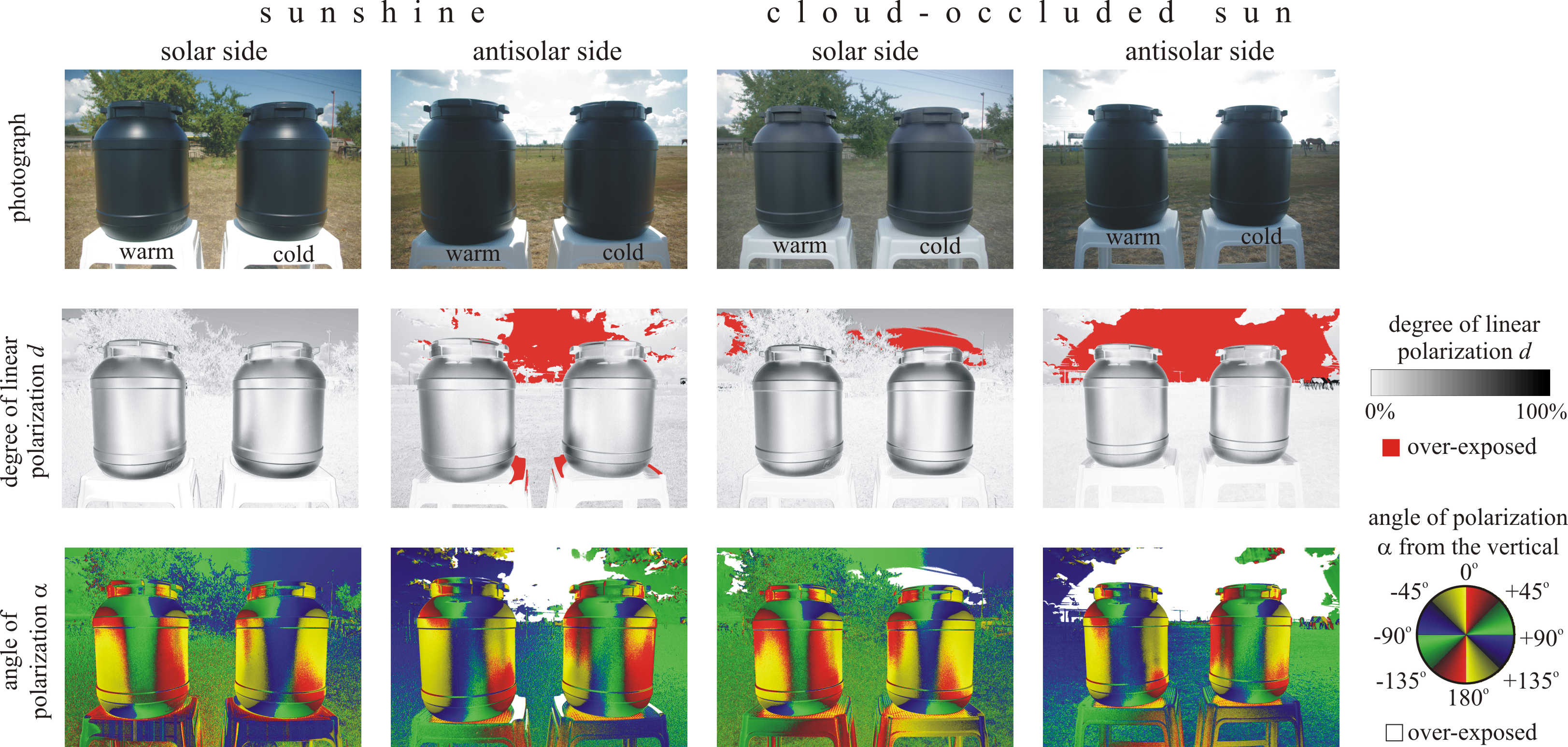


**Supplementary Figure S6:** Colour photographs and patterns of the degree of linear polarization *d* and the angle of polarization α (measured clockwise from the vertical) of light reflected from the solar and antisolar sides of the warm and cold black barrels used in the choice experiments measured by imaging polarimetry in the green (550 nm) part of the spectrum. The polarization patterns were practically the same in the red (650 nm) and blue (450 nm) spectral ranges. In over-exposed pixels at least one of the three (red, green, blue) radiance values reached the maximum (= 255 in true-colour representation).


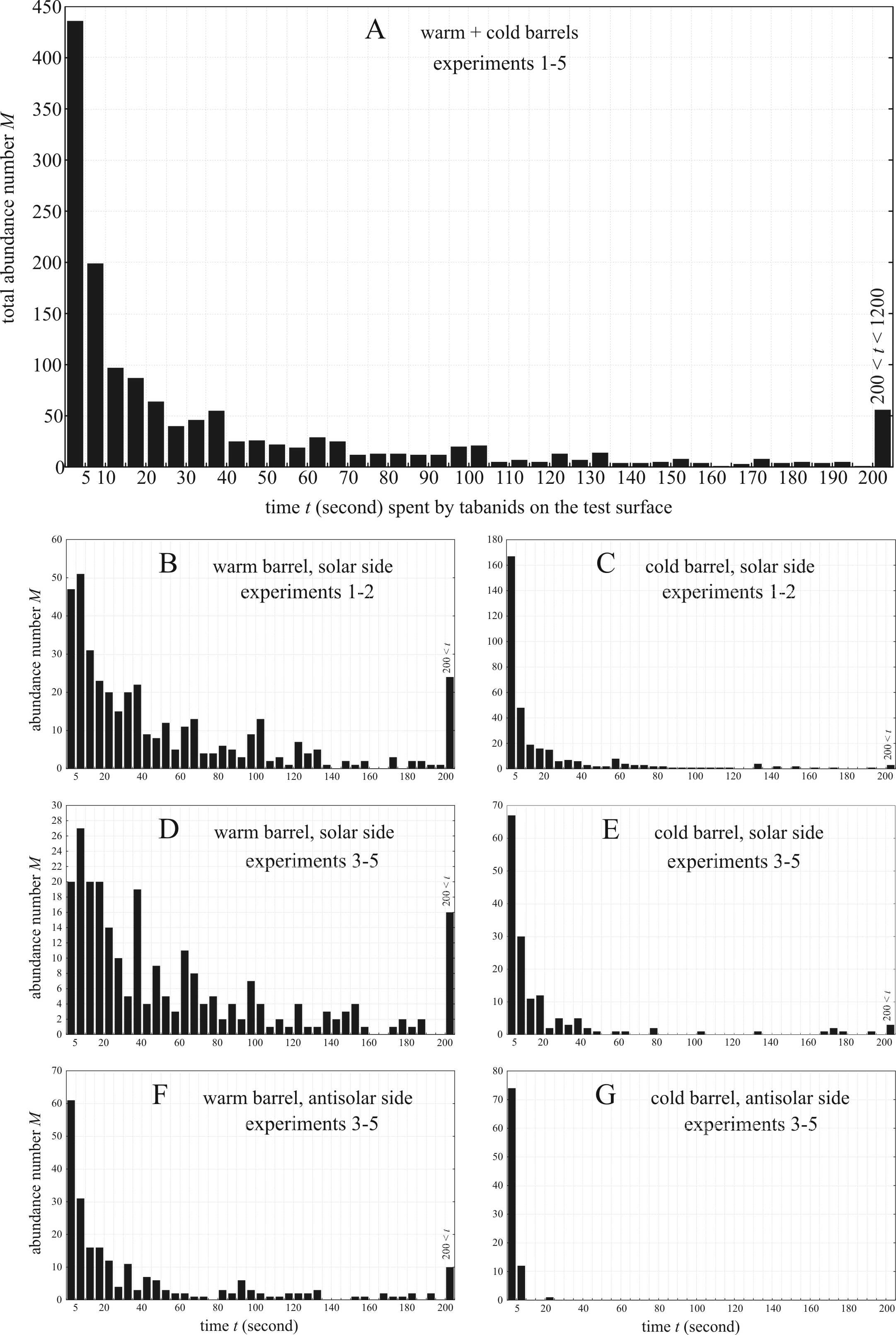


**Supplementary Figure S7:** (A) Total abundance number *M* of time *t* (second) spent by tabanids on the warm and cold barrels in experiments 1-5. The last black vertical column on the right hand side represents time periods 200 s < *t* ≤ 1200 s. (B-C) Abundance number *M* of *t* spent by tabanids on the solar side of the warm (B) and cold (C) barrel in experiments 1-2. (D-G) Abundance number *M* of *t* spent by tabanids on the solar side of the warm barrel (D), on the solar side of the cold barrel (E), on the antisolar side of the warm barrel (F) and on the antisolar side of the cold barrel (G) in experiments 3-5.
